# Complete Genome Sequence of *Paenibacillus polymyxa* CF05, a Strain of Plant Growth-Promoting Rhizobacterium with Elicitation of Induced Systemic Resistance

**DOI:** 10.1128/genomeA.00198-15

**Published:** 2015-04-16

**Authors:** Mengying Lei, Peng Lu, Liping Jin, Yan Wang, Jialing Qin, Xi Xu, Liqin Zhang, Yongjun Wang

**Affiliations:** a Key Laboratory of Forest Protection, College of Forestry and Biotechnology, Zhejiang Agriculture and Forestry University, Lin’An, Zhejiang Province, China; b National Joint Engineering Laboratory of Biopesticide Preparation, Lin’An, Zhejiang Province, China

## Abstract

*Paenibacillus polymyxa* CF05 is a Gram-positive rod-shaped bacterium isolated from the interior of an ancient tree, *Cryptomeria fortunei*, in China. This bacterium displays potent biocontrol effects against certain soil-borne diseases and the elicitation of induced systemic resistance in tomatoes. Here, we report the complete genome sequence of *P. polymyxa* CF05.

## GENOME ANNOUNCEMENT

*Paenibacillus polymyxa*, the type species of the genus *Paenibacillus*, is considered a plant growth-promoting rhizobacterium (PGPR) and is widely used in sustainable agriculture and environmental remediation because of its multiple functions (1, 2). Certain strains of this bacterium, coupled with many plant species, have been developed as biofertilizers or biopesticides used effectively in the control of plant-pathogenic fungi, bacteria, and nematodes (2–4). *P. polymyxa* CF05 was isolated from the interior of an ancient tree, *Cryptomeria fortunei*, on Tianmu Mountain in China (5). This strain is capable of eliciting the induction of the defense response of tomatoes and might be an agent to manage *Fusarium* wilt disease (6). Genome sequencing of *P. polymyxa* CF05 was conducted to obtain additional insights into the physiological characteristics involved in microbe-plant interactions and for future studies examining the molecular basis of these traits.

The completed genome sequence of *P. polymyxa* CF05 was developed at the Beijing Genomics Institute (BGI) (Beijing, China) using Solexa paired-end sequencing technology (7). Genomic DNA was isolated using a DNA extraction kit (DNeasy blood and tissue kit; Qiagen, Valencia, CA). Using total genomic DNA, two gene libraries were prepared from sheared DNA fractions (~500 bp and ~2 kb) using Illumina paired-end sample preparation kits (Illumina, Inc.), according to the manufacturer’s instructions. The DNA was sequenced using an Illumina Solexa GA IIx instrument. The sequencing run yielded 2,887,000 filtered paired-end reads (500-bp insert, 425 Mb in total) and a total of 4,821,000 reads for the 2-kb inserts (394 Mb in total) that were used for deep sequencing. The total sequence data provided 134-fold coverage of the genome. About 97.9% of the reads were assembled into 25 scaffolds with 191 contigs using the SOAPdenovo alignment tool version (http://soap.genomics.org.cn/index.html#intro2). Gaps were filled by Sanger sequencing of the PCR products by custom primer walks and long-distance PCR amplification of the regions between each pair of scaffolds and contigs. The open reading frames (ORFs) were predicted using the NCBI Prokaryotic Genomes Automatic Annotation Pipeline (PGAAP) (8). tRNA and rRNA genes were identified by tRNAscan-SE version 1.3 (9) and RNAmmer version 1.2 (10). The metabolic pathways were examined using the KEGG Automatic Annotation Server (http://www.genome.jp/kegg/).

The completed genome of *P. polymyxa* CF05 consists of a single circular chromosome of 5,762,698 bp, with a G+C content of 45.40%. The origin of replication was identified using Ori-Finder (11). The genome annotation revealed 4,869 coding sequences, 107 tRNA loci, and 43 rRNA operons.

The genes responsible for nitrogen fixation (*nif* genes) are present in the *P. polymyxa* CF05 genome, as well as genes responsible for indole-3-acetic acid synthesis, biomass degradation, antimicrobial production, and volatile organic compound (2,3-butanediol) synthesis. These genes corroborate our results, demonstrating biocontrol, plant growth promotion, and the elicitation of induced systemic resistance (5, 6, 12). Further analysis and study are required to establish the intricacies of this complicated metabolic and signaling pathway for facilitating its application.

### Nucleotide sequence accession number. 

The complete genome sequence of the *P. polymyxa* CF05 chromosome is available in GenBank under the accession no. CP009909.
